# Characterization of the Transcriptome and Proteome of *Brassica napus* Reveals the Close Relation between DW871 Dwarfing Phenotype and Stalk Tissue

**DOI:** 10.3390/plants11030413

**Published:** 2022-02-02

**Authors:** Jing Luo, Sha Huang, Min Wang, Ruimao Zhang, Degang Zhao, Yuanyu Yang, Fang Wang, Zhuanzhuan Wang, Rong Tang, Lulu Wang, Huagui Xiao, Bin Yang, Chao Li

**Affiliations:** 1 Guizhou Oil Crops Institute, Guizhou Academy of Agriculture Sciences, No. 502 Xinzhong Road, Jinxin Community, Huaxi District, Guiyang 550006, China; anrymeng0@hotmail.com (J.L.); hsha33@163.com (S.H.); YYYyuanyu@163.com (Y.Y.); ww18336606772@163.com (F.W.); zhuanzhuan928@163.com (Z.W.); trtwo@126.com (R.T.); wanglulu296@sohu.com (L.W.); xiaohuagui74@21cn.com (H.X.); ybin9855@163.com (B.Y.); 2 School of Life Sciences, Guizhou Normal University, Huaxi University City, Gui’an New District, Guiyang 550025, China; 15692425721@163.com; 3 Guizhou Plant Conservation Technology Center, Guizhou Academy of Agricultural Sciences, No. 502 Xinzhong Road, Jinxin Community, Huaxi District, Guiyang 550006, China; dgzhao@gzu.edu.cn; 4 Guizhou Rapeseed Institute, Guizhou Academy of Agriculture Sciences, No. 111 Duyun Road, Guanshanhu District, Guiyang 520115, China; zhangruimao@126.com; 5 Key Laboratory of Plant Resources Conservation and Germplasm Innovation Center for Mountainous Region (Ministry of Education), Institute of Agro-Bioengineering, College of Life Sciences, Guizhou University, 2708 Huaxi Avenue South Section, Huaxi District, Guiyang 550025, China; 6 Collaborative Innovation Center for Mountain Ecology & Agro-Bioengineering, Institute of Agro-Bioengineering, College of Life Sciences, Guizhou University, 2708 Huaxi Avenue South Section, Huaxi District, Guiyang 550025, China

**Keywords:** *Brassica napus* L., dwarfing mechanism, multiomics, plant height

## Abstract

Rapeseed is a significant oil-bearing cash crop. As a hybrid crop, *Brassica napus* L. produces a high yield, but it also has drawbacks such as a tall stalk, easy lodging, and is not suitable for mechanized production. To address these concerns, we created the DW871 rapeseed dwarf variety, which has a high yield, high oil content, and is suitable for mechanized production. To fully comprehend the dwarfing mechanism of DW871 and provide a theoretical foundation for future applications of the variety, we used transcriptome and proteome sequencing to identify genes and proteins associated with the dwarfing phenotype, using homologous high-stalk material HW871 as a control. By RNA-seq and iTRAQ, we discovered 8665 DEGs and 50 DAPs. Comprehensive transcription and translation level analysis revealed 25 correlations, 23 of which have the same expression trend, involving monolignin synthesis, pectin-lignin assembly, lignification, glucose modification, cell wall composition and architecture, cell morphology, vascular bundle development, and stalk tissue composition and architecture. As a result of these results, we can formulate a hypothesis about the DW871 dwarfing phenotype: plant hormone signal transduction, such as IAA and BRs, is linked to the formation of dwarf phenotypes, and metabolic pathways related to lignin synthesis, such as phenylpropane biosynthesis, also play a role. Our works will contribute to a better understanding of the genes and proteins involved in the rapeseed dwarf phenotype, and we will propose new insights into the dwarfing mechanism of *Brassica napus* L.

## 1. Introduction

*Brassica napus* L. is a Brassicaceae oil-bearing cash crop. Rapeseed is the world’s third-largest edible oil crop and one of Canada, USA, and Germany’s main exported primary products; it is also one of China’s main imported primary products. In 2019, the global import of rapeseed was 5.21 MT, with China accounting for 20.15% [1]. At the moment, there are two main explanations for the origin of *Brassica napus*. The first is that we think *Brassica napus* (AnAnCnCn) originated from heterologous hybridization and polyploidization of *Brassica oleracea* (CoCo) and *Brassica pekinensis* (AoAo) in the European-Mediterranean area [2]; the second is that we think *Brassica napus* originated from different types of *Brassica oleracea* and *Brassica pekinensis* directly hybridize in different locations [3]. In China, the main varieties are hybrid offspring of *Brassica napus*, which was introduced in the mid-twentieth century, and *Brassica pekinensis*, which was introduced from Japan in the 1930s, with winter rapeseed as the main planting type [4].

Rapeseed hybrid breeding is the primary method for increasing rapeseed oil yield. From 1985, when the first three-line rapeseed hybrid Qinyou NO.2 was bred, to the present, hybrid varieties have accounted for 60% of global planting area, with China accounting for 75% [5,6]. Continuous breeding of hybrid rapeseed has not only increased yield but also plant height. The main cause of rapeseed lodging is top-heaviness caused by tall plants, which is manifested by varying degrees of bending and breaking of the stalk [7]. Lodging increases plant morbidity, delays harvest, reduces quality and yield, and impedes mechanized production [8]. As a result, breeding dwarf varieties that are resistant to lodging and suitable for mechanized production has become breeders’ primary focus. Williams and Hill were the first to report dwarf rapeseed mutants. They discovered two dwarf mutants, which they named *Bn5-2* and *Bn5-8* [9]. Shi et al. obtained dwarf mutants *ds-1* and *ds-2* by treating breeding materials with 0.25% EMS (Ethylmethylsulfone) in China [10].

**Figure 1:**
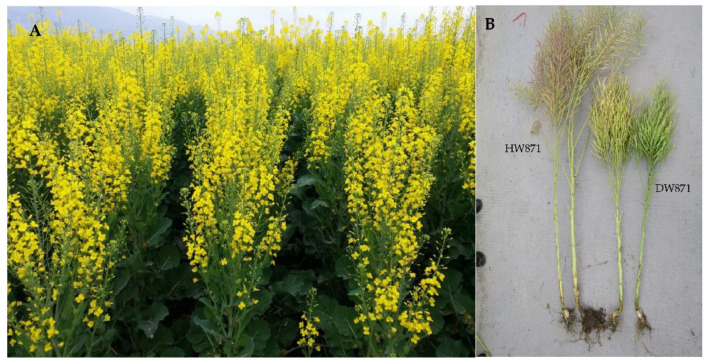
(**A**) DW871’s actual planting situation; (**B**) comparison of DW871 and HW871 siliques.

DW871 is a new semidwarf *Brassica napus* variety with outstanding characteristics [11]. According to various planting methods, the average plant height ranges from 70 cm to 139.1 cm, the harvest index is 0.37, and the silique density is 1.8 cm (Figure 1). In comparison to high variety, its thicker xylem keeps the plants upright throughout the growth period, the firm fruiting branches ensure that the siliques do not fall off due to external factors such as inclement weather, and the inflorescence and inflorescence segment are both significantly different from other rapeseeds varieties that have been put into production. We chose DW871 as the research object for this study. The genes and proteins that play a significant role in the development of the dwarfing phenotype are identified using a combined transcriptome and proteome analysis. It also explained how the DW871 dwarf type is produced and provided a reference for the location of the DW871 dwarf gene.

## 2. Results

### 2.1. Plant Height and Inflorescence Length

From the bolting stage, we measured plant and inflorescence heights. By observing the trend of plant height and inflorescence length for 20 days. We discovered that the growth rate of plant height and inflorescence length of DW871 and HW871 show a decreasing trend. In 10 and 20 days, the DW871 plant height elongated by 15.32 cm and 0.81 cm, respectively, and the DW871 inflorescence length elongated by 16.51 cm and 3.04 cm; the HW871 plant height elongated by 45.87 cm and 20.23 cm, respectively, and the HW871 inflorescence length elongated by 6.38 cm and 15.39 cm. This indicates that both the DW871 and HW871 plant height elongation rates decreased, but in terms of inflorescence length, the DW871 elongation rate decreased while the HW871 elongation rate increased. Finally, DW871 was 93.19 cm shorter than HW871 and the inflorescence segment of DW871 was 15.71 cm longer than HW871 (Table 1).

### 2.2. Stalk Tissue

**Figure 2:**
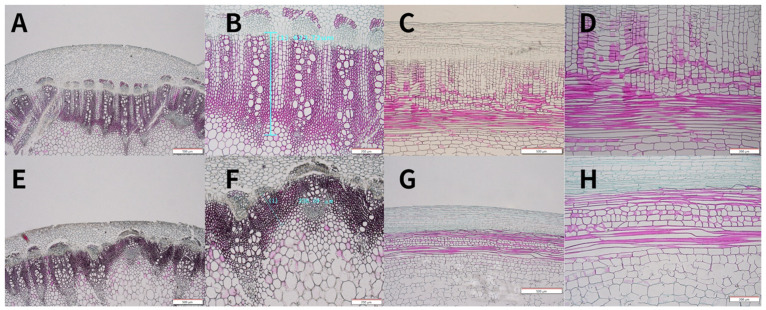
Paraffin sections of stems DW871 (**A**–**D**) and HW871 (**E**–**H**). (**A**,**E**): transverse section of stems (40×); (**B**,**F**): transverse section of parenchyma; (**C**,**G**): radial section of stems (40×); (**D**,**H**): radial section of parenchyma (200×).

The height of a plant is influenced by a variety of factors. At the cellular and tissue levels, these factors are linked to changes in cell morphology and tissue structure. Plant height, in particular, is closely related to changes in stalk tissue structure. This study used paraffin sections of DW871 stalk tissue to explain the causes of DW871 dwarfing at the cellular level. The transverse section results show that the thickness of the DW871 cortex increased when compared to HW871; the thickness of the cambium increased, and the number of cambium cells increased; the cambium was approximately semicircular; the parenchyma was more neatly arranged, the number of vascular bundles increased, and the boundary between the pith and parenchyma cells was more obvious (Figure 2A,E). The thickness of DW871 xylem was 623.72 m, while the thickness of HW871 xylem was 326.59 m, a difference of 1.91 times. The vertical section results show that when DW871 was compared to HW871, the parenchyma cells had similar morphology (Figure 2C,G), the length of the parenchyma cells shortened in the vertical direction, but the number of parenchyma cells increased in the horizontal direction (Figure 2D,H).

### 2.3. Transcriptomic Analysis

**Figure 3:**
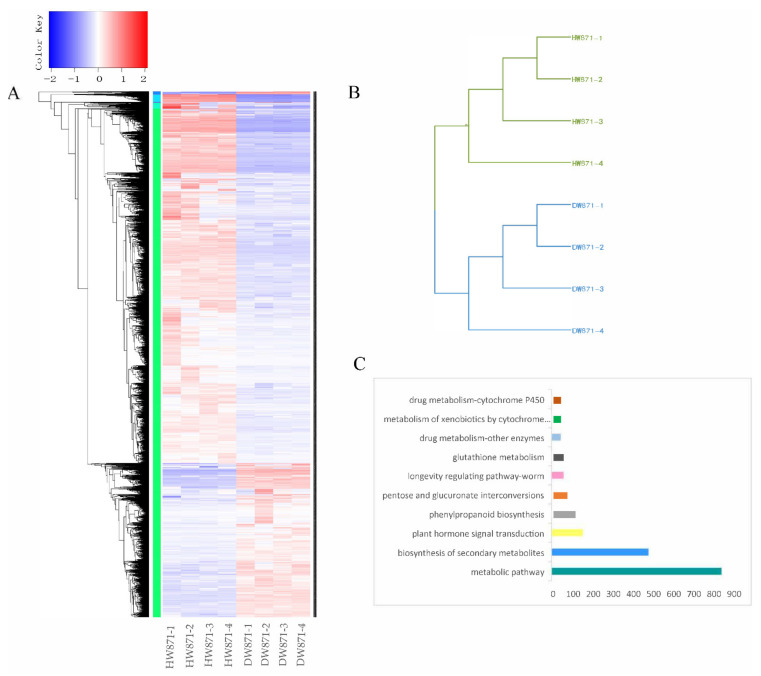
(**A**) DEGs cluster; (**B**) clustering results between samples; (**C**) KEGG pathway.

Using illumia sequencing, a total of 275.81 UMI reads were generated, resulting in the assembly of 10141 genes. We identified 8665 DEGs between DW871 and HW871 by FPKM value, which included 2582 upregulated and 6083 downregulated DEGs (Figure 3A). The gene expression profile of biological repeats from the same group showed a high correlation coefficient, whereas samples from different groups showed a relatively low correlation coefficient (Figure 3B).

DEGs were annotated in 295 KEGG pathways and 264 GO terms. The KEGG pathways with the highest DEGs were metabolic pathway (40.83%), biosynthesis of secondary metabolites (23.32%), plant hormone signal transduction (7.5%), phenylpropanoid biosynthesis (5.37%), pentose and glucuronate interconversions (3.43%), longevity regulating path-way-worm (2.85%), glutathione metabolism (2.52%), drug metabolism-other enzymes (2.23%), metabolism of xenobiotics by cytochrome P450 (1.89%), and drug metabolism-cytochrome P450 (1.89%) (Figure 3C).

### 2.4. Identification of DAPs by iTRAQ

**Figure 4:**
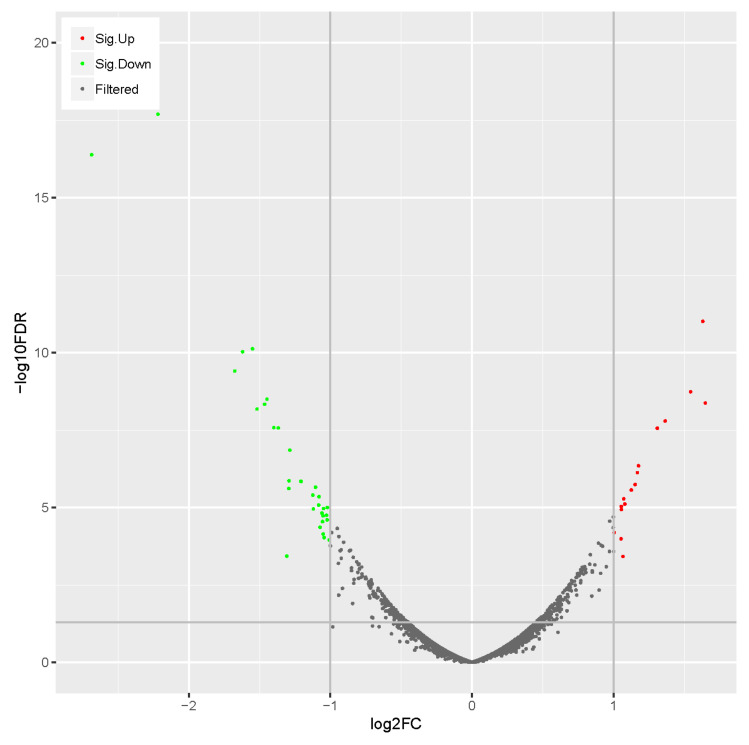
Volcano of DAPs. X axis represents the logarithm of the differential expression level of genes when the base is 2; Y axis represents the logarithm of FDR (false discovery rate) when the base is 10; red, green and black dots represent up- and down- and nonregulated DEGs respectively.

To elucidate the mechanism of dwarf phenotype formation, we used comparative proteomics to detect DW871 and HW871. Protein sequencing from dwarf mutant and wild-type samples yielded 600743 spectra, 39669 of which were unique peptides. They were able to identify 5839 proteins. Even though most protein expression differences between dwarf mutant and wild-type are indistinguishable, we identified 50 DAPs, with 16 upregulated proteins and 34 downregulated proteins (Figure 4).

We used the GO database to analyze and annotate biological and biochemical functions in order to obtain functional information about identified proteins. We annotated 24 GO terms with FDR ≤ 0.05. In the biological process, response to stimulus (60.6%) enriched the most proteins, followed by response to stress (57.6%), defense response (33.3%), and interspecies interaction between organisms (30.3%). In the cellular position, cell wall (33.3%) and external encapsulating structure (33.3%) were enriched by the same amount of protein, followed by apoplast (21.2%) and extracellular region (21.2%), which were enriched to the same amount of protein.

**Figure 5:**
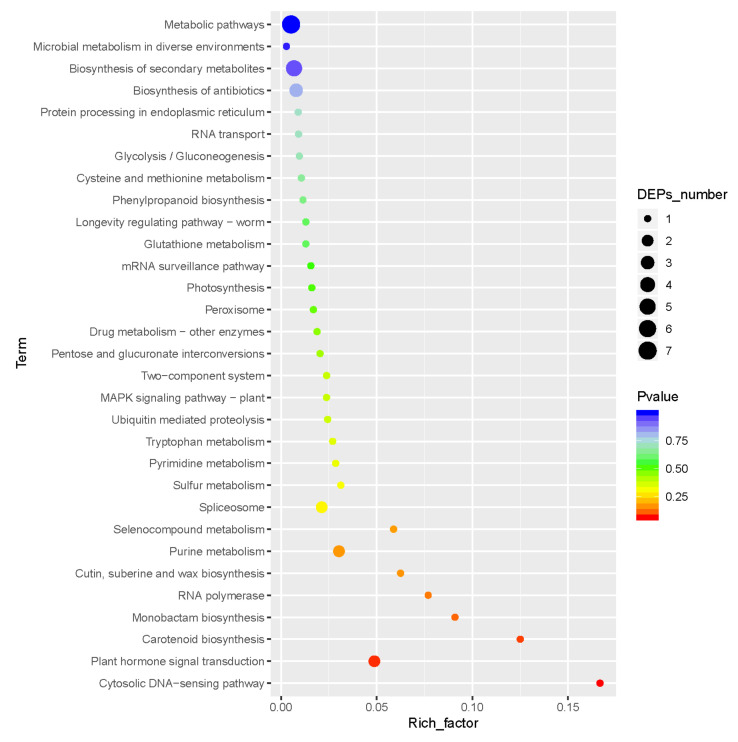
KEGG enrichment of DAPs. X axis represents enrichment factor, Y axis represents pathway name; the change in color from blue to red is the translation level of the protein annotated to the pathway. The redder the color, the bigger the gap. The size of the dot indicates the number of DAPs annotated to the pathway.

Among the 50 DAPs, 17 DAPs had a KEGG pathway annotation that matched. DAPs are enriched in 31 KEGG metabolic pathways (Figure 5). The top ten KEGG pathways are: cytosolic DNA-sensing pathway (16.67%), carotenoid biosynthesis (12.5%), monobactam biosynthesis (9.09%), RNA polymerase (7.69%), cutin suberine and wax biosynthesis (6.25%), selenocompound metabolism (5.88%), plant hormone signal transduction (4.88%), sulfur metabolism (3.12%), purine metabolism (3.03%), and pyrimidine (2.86%).

### 2.5. Correlation Analysis of Proteomic and Transcriptomic Results

**Figure 6:**
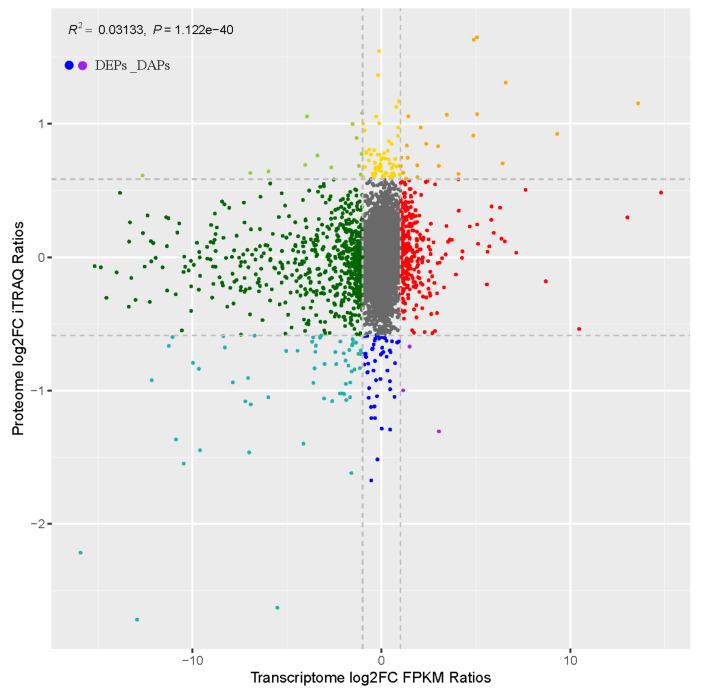
The correlation between DEGs and DAPs. DEPs _ DAPs represent the point where there is a correlation between DEGs and DAPs, R represents the correlation coefficient, and *p* value represents the significance level.

To clarify and explain the mechanism underlying dwarfism. We compared the proteome and transcriptome results to see if changes in translational levels are related to changes in related transcripts. When a protein has a matching RNA, we think there is an association between the protein and the RNA. We found 50 proteins with corresponding genes (correlation coefficient *r* = 0.1770) (Figure 6).

**Figure 7:**
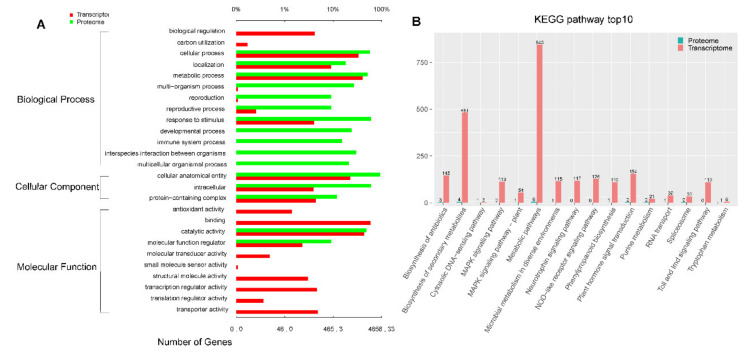
(**A**) GO enrichment analysis for DEGs and DAPs; (**B**) top 10 pathways for KEGG enrichment.

Furthermore, we identified 25 common correlations from 50 DAPs in proteomic analysis and 8665 DEGs in transcriptomic analysis. We identified metabolic and signal transduction pathways that are closely related to the dwarf phenotype in order to further investigate correlations using KEGG and GO enrichment analyses. DAPs and DEGs were significantly enriched in defense response, stress response, and external encapsulating structure for GO enrichment (Figure 7A). DAPs and DEGs were significantly enriched in pentose and glucuronate interconversions, peroxisome, external encapsulating structure, plant hormone signal transduction, and carotenoid biosynthesis in terms of KEGG enrichment (Figure 7B).

### 2.6. Correlation Analysis of DAPs and DEGs with Same Changing Trend

There is a complex nonlinear relationship between mRNA and proteins, rather than a simple correspondence. The 25 correlations are classified into two groups based on the changing trends of DAPs and DEGs (Table 2): (1) DAPs and DEGs are upregulated or downregulated, and (2) DAPs are upregulated but DEGs are downregulated, or vice versa. The correlations between groups 1 and 2 are 23 and 2, respectively. Analyzing DAGs and DEPs with the same changing trend can help to highlight the differences between the two omics and further investigate the reasons for the differences at the molecular level. As a result, we examined 23 correlations with the same changing trend.

Except for two opposite correlations (BnaAnng37980D, BnaC03g30640D), 23 correlations have the same changing trend. There is research that has predicted that BnaC03g30640D might participate in the formation of plant vascular tissue. The other 23 correlations can be divided into four groups: (1) metabolism-related genes: XET, CEI, BG1, LOX, SEP, and AEP, among others, endopeptidases are all upregulated, while other correlations are all downregulated; these correlations involve lignin and pectin degradation, fatty acid metabolism, and protein hydrolysis; (2) defense and stress response: thaumatin, SD, DES, Bet 4 1, PHI1, and ER transmembrane protein are all downregulated, possibly due to external pressure; (3) transmembrane transport of substances: nsLTPs, AAT, and FAR play an important role in the transmembrane transport of hormones and signals between cells, as well as the regulation of transmembrane transport channels by cells; (4) transcription: RNA binding is important in the process of protein transcription and translation.

### 2.7. Highly DEGs in Plant Hormone Signal Transduction Pathway

Plant growth is a physiological process that is influenced by a variety of factors, and plant hormones play an important role in it. Plant hormones are involved in the process of maintaining apical dominance and shaping the plant shape, particularly for plant height traits. There are 155 DEGs and 2 DAPs that have been annotated. We consider the role of auxin and BRs (brassinosteroids) in plant growth, especially in stalk elongation. We further analyzed the DEGs and their expressions in the above-mentioned hormone-related signal transduction pathways.

AUX1 (auxin influx carrier) participates in polar auxin transport between cells and regulates plant development by regulating auxin transmembrane transport. The DEG encoding AUX1 is downregulated by 3.63 times. In the BRs signal transduction pathway, XET regulates cellular elongation, their expressions are downregulated, and two encoding genes are downregulated by 3.92 times and 5.42 times respectively. It’s indicated that compared with HW871, DW871 is more active in IAA, and the activity of BRs signal transduction pathway is inhibited.

### 2.8. Highly DEGs in Phenylpropane Biosynthesis

There are two important pathways in phenylpropane biosynthesis: monolignol biosynthesis and flavanone biosynthesis. In the monolignol biosynthesis, the metabolites contain S-, G-, and H-lignin. We analyzed 22 DEGs which involve in the monolignol biosynthesis. They are coded into PAL, CYP73A, 4CL, HCT, CCR, CAT, COMT, and CCoAOMT (Table 3). Except for one gene about PAL, COMT, and CCoAOMT, all DEGs were up-regulated.

### 2.9. Highly DEGs in Phenylpropane Biosynthesis

In order to validate the reliability of transcriptome sequencing results. We measured and analyzed the expression of labeled DEGs in transcriptome sequencing to validate the sequencing results by real-time qRT-PCR.

**Figure 8:**
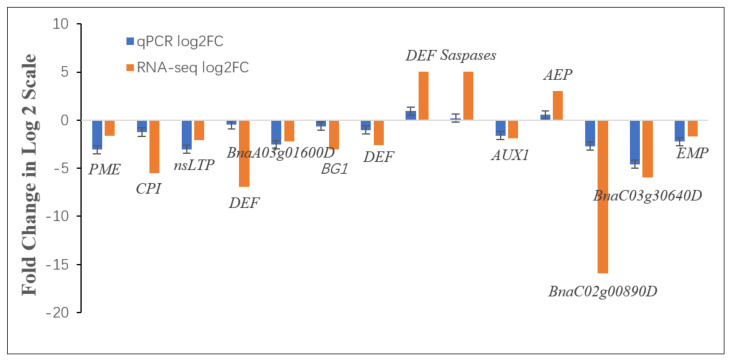
DW871 significant difference gene qRT-PCR verification. Ordinate shows the logarithm of differential multiples of the corresponding gene, and the positive and negative values of y axis express the gene up or down, respectively.

We selected 14 genes from metabolic pathways that are highly enriched and closely related to cell elongation and plant growth to design primers, and performed qRT-PCR. The results show that the above 14 genes’ qRT-PCR results are consistent with the transcriptome sequencing results’ expression trends (DW871 vs HW871) (Figure 8). These results indicate that the RNA-seq data in the present study are reliable.

## 3. Discussion

### 3.1. Metabolic Genes Are Involved in the Construction of Plant Architecture

The observation results of plant growth show that, compared with HW871, DW871 displayed dwarfism, faster lignification, and longer inflorescence. These results confirm the significant differences in metabolic progress between the two. The results of microscopic observation show that the changes in stalk tissue structure and stalk cell morphology are important factors leading to dwarfing. In summary, these results show that the formation of DW871 dwarfing traits is closely related to changes in metabolic genes expression.

PE is the rate-limiting enzyme in pectin degradation and is essential for cell wall formation and degradation [12]. It can also control the mechanical strength and elasticity of the cell wall, as well as the lignification of the stalk tissue [13,14,15]. Both their underexpression and overexpression can cause flaws in the pectin-lignin complex assembly [16]. The total downregulation of PE may change the degree of methyl esterification, improve stalk mechanical strength and cell wall strength, inhibit cell wall degradation, and finally inhibit DW871 growth. This corresponds to the actual observation.

BG1 is a member of the carotenoid biosynthesis pathway, and it plays a role in catalyzing ABA-D-glucopyranosyl ester (ABA-GE) synthesis, glycosidic bond hydrolysis, transglucosylation progress, and cyanogenic glucoside synthesis [17,18,19]. Prior research has shown that BG1 can participate in lignification by regulating the release of monolignins, as well as affect plant growth rate by regulating the concentration of cyanogenic glucosides in plants [20,21]. The downregulation of the three genes encoding BG1 may be related to the lignification of stalk tissue.

Plant LOXs primarily catalyze the conversion of polyunsaturated fatty acids like linolenic acid and linoleic acid into unsaturated fatty acid hydrogen peroxide and other easily stored substances, and they exist as storage proteins [22]. According to recent research, LOXs can act as signal molecules to activate jasmonic acid signal transduction in plant defense and stress response [23]. Furthermore, Kolomiets et al. discovered that LOXs are highly active in potato tubers, playing a role in tuber growth, expansion, and division of parenchyma cells [24]. The downregulation of genes encoding LOXs may be the cause of the changes in the parenchyma structure.

In summary, there are numerous significant differences between DW871 and HW871, including pectin-lignin assembly, cell wall component and architecture, lignification, and glucose modification. A higher degree of methyl esterification and tighter intercellular architecture give DW871 greater mechanical strength, which may inhibit stalk cell elongation and division; changes in monolignin component and lignification progress lead to changes in plant growth progress.

### 3.2. Defense and Stress Response May Play a Key Role in Plant Height

All rapeseed used in this study came from the Chinese winter oilseed rape area. After low winter temperatures, rapeseed will complete vernalization around mid-February and enter a period of rapid growth. DW871’s overall growth rate is faster than that of HW871 during this period, and it enters the initial flowering stage before HW871, which may be due to differences in their adaptability to the environment. In order to explain this phenomenon, we identified seven DEGs/DAPs in defense and stress response. thaumatin-like proteins (TLPs) are involved in much important biological progress, such as Stress response to fungi and low-temperature response, and cell wall modification, and programmed cell death [25]. FAR mainly regulates drought-resistant and water-saving, by synthesizing initial tax, and plays a role in the formation of corkification and lignification [26,27]. SD can inhibit the activity of insect sodium channels and contribute to pest defense mechanisms. In the past reports, researchers thought PHI1 is mainly involved in a brassinosteroid-dependent regulatory pathway that controls growth and development under low carbon and energy availability [28]. But in recent reports, the overexpression of PHI1 makes the transgenic plants show higher, longer stalk tissue fibers, and smaller and denser xylem cells [29].

Thus, significant changes in TLPs, as well as down-regulation of FAR, SD, and PHI1, may result in changes in environmental adaptability, stalk component and structure, cell size, and eventually result in a dwarf and highly lignified phenotype.

### 3.3. Transport Proteins Regulate Plant Height by Controlling Material Transport

Transport proteins mediate the transport of specific substances on both sides of the biofilm, and thus play a role in almost all cellular actions such as nutrient intake, metabolite release, and signal transduction.

Plant growth or changes in height mainly depends on the division of cells in the meristem region and the growth of cells in the elongation region. And the main factor restricting cell growth in the elongation region is the binding effect of the cell wall. Cell wall-binding proteins can regulate the growth of plants by regulating the activity of the cell wall expander protein and changing the extensibility of the cell wall [30]. Because of their complex sequence diversity, LTPs are thought to be functionally diverse [31]. In plants, LTPs are generally supposed to be involved in the in vitro transport of lipids and deposition of keratin. The mechanism model of LTPs regulating cell wall-loosening shows: LTPs can be associated with the cellulose-xyloglucan network and can interfere with the network in the cell wall matrix. In addition, Nieuwland presumed LTPs is involved in nonaqueous degradation of cell walls and regulate the extensibility of cell wall [32]. The downregulation of LTPs results in cell wall extension inhibition and cell elongation inhibition, causing the entire plant to be dwarfed. This is supported by microscopic observations.

Plant ER can control the modification of polysaccharides and the composition of the cell wall to achieve the purpose of regulating the elongation of the cell wall [33]. When the Arabidopsis ER transmembrane protein is misexpressed, Arabidopsis and its control group exhibit significant differences, including defects in cell elongation, abnormally thickened secondary walls, and extreme dwarf [34]. This is consistent with the observation that DW871 has a higher lignification degree, a relatively short plant architecture, and the obstruction of longitudinal cell elongation. It may explain the relationship between the downregulation of ER transmembrane protein and dwarf.

Taken together, changes in cell wall elongation and material composition may cause DW871 to be dwarfed.

### 3.4. The Changes in Lignin Synthesis is an Important Factor of Dwarf

**Figure 9:**
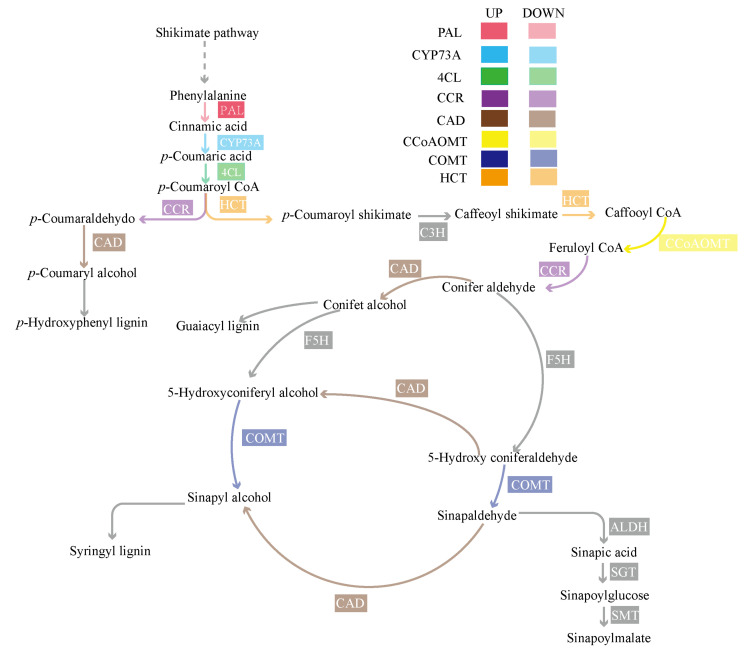
Monolignol synthetic pathway.

The foregoing results supposed the important relationship between dwarf and cell wall extension and lignification. It indicates that lignin may play a role in dwarfing. To further reveal the relationship between lignin synthesis and its component and dwarfing phenotype, we analyzed the pathway of phenylpropane biosynthesis (Figure 9). Phenylpropanoid biosynthesis includes the common pathway and the specific pathway [35].

In the common pathway, PAL is the rate-limiting enzyme, and plays a role in regulating lignin synthesis [36]. PAL could participate in cross-linking between arabinoxylan or arabinoxylan chains and lignin, and strengthen cell wall hardness [37]. Moreover, PAL could eliminate ROS generated during lignification to enhance cell wall strength [38]. When Arabidopsis PAL activity is reduced, the plant exhibits growth retardation and dwarfing phenotypes [39]. Our transcriptomic analysis results identified PAL encoding unigenes, such as BnaA05g28470D, which exhibits PAL activity and is upregulated, and BnaC05g42780D, which exhibits PAL inhibitor activity and is downregulated. These results show that lignin synthesis will become more active, which is supported by microscopic observations. HCT functions as a dual-activity acyltransferase, catalyzing the synthesis of shikimate/quinic acid and p-coumarin-CoA to 4-coumaroyl shikimate/quinic acid. In past research, the downregulation of HCT will cause the loss of GA sensitivity, and may cause a decrease in cell elongation [40]. Our results indicated that the genes encoding HCT are downregulated, which might indicate that HCT participates actively in the formation of dwarfing phenotype.

In specific pathways, CCR, CAD, and COMT as key enzymes are involved lignin synthesis [41]. In plants, the deletion or expression change of any of the above single enzymes will result in a significant change in phenotype [42,43,44]. When CCR and CAD are both deleted, Arabidopsis will appear dwarfing phenotype and male sterility [45]. When the expressions of CCR, CAD, and COMT are inhibited, tobacco will exhibit a dwarfed phenotype and slow growth [46]. In this study, the expression of CCR, CAD, and COMT were down, but the growth of the plant was not delayed. We believe that DW871 has an unknown regulatory mechanism that causes plant dwarfing without affecting growth rate.

### 3.5. IAA and CTK-Mediated Plant Architecture Changes

The different periods of plant growth and development involve various plant signal transduction. The transcriptome and proteome results revealed that there are very few DAPs that are annotated in plant signal transduction. This shows that changes in signal transduction mainly occur at the transcript level instead of the translation level. As a sensitive hormone, changes in auxin synthesis and auxin signal response will lead to changes in plant growth.

IAA was the first plant hormone to be discovered. IAA as a sensitive hormone, changes in auxin synthesis and auxin signal response will cause changes for plant growth. AUX1 is the member of amino acid/auxin permease (AAAP) and regulates the polar transport of auxin. At the moment, there are mainly two presumptions about auxin regulating plant growth: (1) auxin binds to the H+ ATPase on the cell membrane, causing H+ to be transported to the outside of the cell and increasing the pH in the intercellular environment, and (2) auxin regulates cell wall softening and expansion [47,48]. Even so, no matter what the presumption is, the participation of AUX1 is required. Our transcriptomic analysis results showed five AUX1-encoding DEGs, and indicated that the obstruction of the transport of auxin into the cell caused decreasing in auxin signal strength and obstruction of the cell wall-extension.

As the strength of hormone signals varies, plant growth processing will also change. In previous studies, inhibiting auxin synthesis and transport resulted in a decrease in the activity of PE, LOXs, PHI1, PAL, and HCT, thereby affecting plant growth [29,40,49,50,51]. There are some distinctions between PAL and other enzymes among them. When the auxin signal strength was reduced, the activity of PAL did not decrease immediately but remained constant for a period of time, which could explain why DW871 did not show growth retardation [52].

BRs are important in cell elongation and division, vascular bundle differentiation, changes in leaf morphology, plant fertility, cell senescence, and stress response [53,54]. BRs eventually activate XET to regulate cell elongation after a series of signal transmissions [55]. XET is primarily involved in the development of xylem and secondary phloem cell walls, xylem cell expansion, lignification progress, and internode growth [56]. The fact that the two DEGs encoding XET are downregulated may indicate that cell elongation is one of the important factors in dwarfism.

## 4. Materials and Methods

### 4.1. Plant Materials and Cultivating Condition

**Figure 10:**
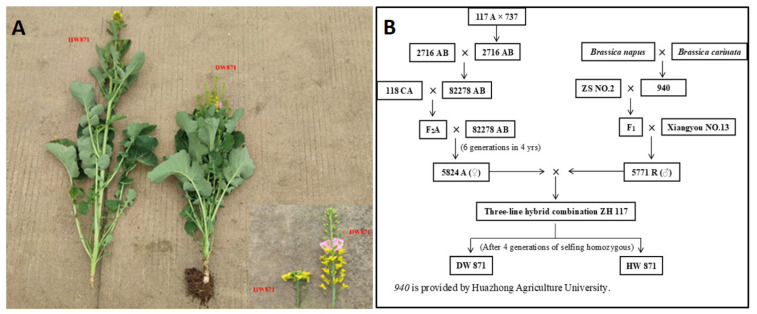
*Brassica napus* L. HW871 and DW871, the small picture shows the inflorescence segment (HW871: left, DW871: right) (**A**); Breeding process of DW871 and HW871(**B**).

In our experiments, Guizhou Rapeseed Institute provided *Brassica napus* L. dwarf variety DW871 and control group high variety HW871 (Figure 10A). We used *Brassica napus* varieties 940, ZS NO.2, and Xiangyou NO.13 to compound cross in many years of hybridization experiments and in the hybrid offspring, we obtained material "5771 R". In subsequent hybridization experiments, we used 5771 R as the male parent and 5824 A as the female parent to create the recessive nuclear sterile three-line hybrid ZH 117. In 2008, we discovered a mutant of the compact dwarf type. After four generations of selfing, we were able to obtain Brassica napus dwarf material (named "DW871") and a homologous tall stalk line (named "HW871") that could inherit plant height traits in a stable manner (Figure 10B).

The test materials came from Tangtou Town, Sinan County, Guizhou Province, China. The latitude and longitude of Tangtou Town are 27°41’ N and 108°6’ E, the altitude is 418 m, and there is a humid subtropical monsoon climate. In the growth period (2019.10–2020.3), the average temperature is 11.96 °C, the max and min temperature are 34.89 °C and 1.22 °C respectively. The precipitation is 346.72 mm, and the sunshine duration is 346.33 h. Compared with the past, the sunshine duration is lower, the precipitation is higher, and the average temperature is similar.

We chose the fourth-to-last internode as the experimental materials to ensure the experiment’s reliability and accuracy. We also established four biological repeats. The internodes were rapidly frozen in liquid nitrogen before being stored at –80°C. Finally, the samples were sent to Shanghai Biozeron for sequencing analysis.

### 4.2. Plant Traits and Stalk Tissue Section

There are significant differences between plant height and inflorescence. We selected plants with similar growth, recorded the plant height and inflorescence length every 10 days from the bolting period, and calculated the arithmetic mean.

We handled internodes in accordance with paraffin slicing. The stalk tissue section was 4 µm. We recorded the cross-section and longitudinal section of the plant tissue.

### 4.3. RNA Extraction and Sequencing

Using the TRIzol® Reagent kit (Invitrogen, Carlsbad, CA, USA), total RNA was extracted from rapeseed stalk tissue. The purity and concentration of RNA were determined using NanoDrop (Invitrogen, Carlsbad, CA, USA) and Qubit (Invitrogen, Carlsbad, CA, USA), respectively, and the integrity of the RNA was determined using 1% agarose gel electrophoresis. The library was then built using the Illumina TruseqTM RNA sample prep kit method. To begin, 1 µg of total RNA was extracted from each sample using the TruseqTM RNA sample prep Kit (Thermo Fisher Scientific, Bedford, MA, USA), and then the mRNA was isolated using the magnetism pearl method and interrupted by ions. The mRNA fragments were reverse transcribed using random hexamer primers, and then double-stranded cDNA was synthesized. The double-stranded cDNA was then processed, which included end-filling, adding A to the 3’ end, and ligating index adapters. The cDNA fragments with adapters were amplified by PCR. The band containing the target fragment was recovered using a 2% agarose gel. According to the manufacturer’s instructions, the quantification was carried out on a TBS380 Picogreen (Invitrogen, Carlsbad, CA, USA), and the qualified library was amplified on cBot (Thermo Fisher Scientific, Bedford, MA, USA) to generate the cluster on the flow cell. After cluster generation, the libraries were sequenced on an Illumina Novaseq 6000 platform, according to the manufacturer’s instructions, and paired-end reads were obtained.

### 4.4. Transcriptomes Data Analysis

High-quality data are required for accurate results. Before analysis, trimmomatic software was used to remove low-quality data by (1) identifying adapter data in reads; (2) removing reads that had no inserted fragments as a result of adapter self-ligation; (3) removing bases with a quality value less than 20 at the 3’ end; (4) when there are bases in the sequence with a quality value less than 10, removing the entire sequence; (5) removing reads with an N ratio of more than 10%; (6) after removing the adapter and quality trimming, removing any sequences that were less than 70 bp in length. After obtaining high-quality clean reads, the clean reads were aligned with the *Brassica napus* reference genome using Hisat2 software.

### 4.5. Protein Extraction and iTRAQ

After prechilling the samples at –20°C, we deep-froze them at –80°C. According to the introduction, the samples were quickly ground into powder under liquid nitrogen, and total protein was extracted using the Plant Total Protein Extraction Kit (Sangon Biotech, Shanghai, China). After quantifying the protein on a Nanodrop 2000c (Thermo Fisher Scientific, Bedford, MA, USA), 200 µg of protein was taken, 5 µL of 1M DTT solution was added, mixed, and incubated at 37 °C for 1h; 20 µL of 1M IAA solution was added, mixed, and the reaction was performed in the dark at room temperature for 1h. We pipetted all of the samples into ultrafiltration tubes, centrifuged them and discarded the supernatant liquor. We added 100 µL UA (8 M urea, 100 mM Tris-HCl, pH8.0) to the ultrafiltration tube, centrifuged, and discarded the supernatant liquor; we repeated this twice. We added 100 µL of 0.5M TEAB, centrifuged, discarded the supernatant liquor, and repeated this process three times. We replaced the collection tube, added trypsin to the ultrafiltration tube in a 50:1 protein-to-enzyme ratio, and performed enzymolysis in a 37 °C water bath for 12–16 h.

We took about 100 µg of each peptide group. Peptides were labeled using the iTRAQ Reagent 8Plex Multiplex Kit (AB SCIEX, Redwood City, CA, USA), according to the manufacturer’s instructions. Peptides with different labels were collected and desalted using a Strata X C18 column (Phenomenex, Torrance, CA, USA), then vacuum centrifuged to dry. Peptides were separated using an Easy nLC/Ultimate 3000 (Thermo Scientific, Massachusetts, MA, USA) and a C18 1.9 mm 150 µm × 120 mm (Thermo Scientific). Peptides were separated at 1 mL/min with a gradient of 5% Buffer B (5% H2O, 95% ACN, pH 9.8) for 10 minutes, 5–35% Buffer B for 40 minutes, and 35–95% Buffer B for 1 minute. The sample was then kept in 95% buffer B for 3 minutes before being changed to 5% buffer B for 1 minute and equilibrated with 5% buffer B for 10 min. Elution absorbance was measured at 214 nm and collected every 1 minute. The peptides were separated into 20 groups and vacuum dried.

### 4.6. HPLC Classification and LC-MS/MS Analysis

Using 100 mL flow phase to dissolve protein samples, we vortexed the protein solution and centrifuged it at 14,000 *g*/min for 20min, and then took the supernatant. We set up 60 blank centrifuge tubes, labeled No. 1-60. We used C18 chromatographic column for separation, according to times of 3, 5.1, 10, 35, 45, 53, and 58 min. The ratio of water to acetonitrile was 97:3, 97:3, 95:5, 82:18, 66:34, 5:95 and 5:95, the flow rate was 700 µL/min. Starting from 5 minutes, we collected the eluate every 1.5 minutes and put it into the labeled centrifuge tubes in turn. After vacuum freeze-drying, it was reconstituted with 0.5% FA. We combined 60 centrifuge tubes into multiple components, centrifuge at 14000g/min for 10 min. We aspirated the supernatant and marked and graded the samples.

The sample after HPLC fractionation was equilibrated with 95% mobile phase A. The sample protein was analyzed on the mass spectrometry platform Q Exactive Plus. The injection conditions were as follows: (1) time: 0, 1, 45, 55, and 60 min; (2) the ratios of flow phase A and flow phase B were 97:3, 94:6, 72:28, 62:38, 1:99 and 1:99; and (3) the flow rate was 300 µL/min.

### 4.7. Protein Data Processing

After the protein samples were fractionated by HPLC and analyzed by LC-MS/MS, we used the database 449905-X101SC20080461-Z01-UniProt-Brassica+napus.fasta (Proteome Discover 2.2) to compare the protein data. In addition, the quality of the protein group was evaluated by referring to the peptide length parameter of the protein group, and distributions of the parent ion mass tolerance distribution, the unique peptide number, the protein coverage, and the protein molecular weight.

### 4.8. Connection of Transcriptome and Proteome

Before connecting the transcriptome and proteome data, it was necessary to screen DAPs (differentially accumulated proteins) and DEGs (differentially expressed genes), the conditions were as follows: (1) FC (Fold Change) ≥ 2 or FC ≤ 0.05; (2) *p* (*p* value) < 0.05. We believe that the DAPs or DEGs are upregulated when FC ≥ 2, *p* < 0.05, the DAPs or DEGs are downregulated when FC ≤ 0.05, *p* < 0.05. In order to calculate the expression level of DAGs and DEPs, and eliminate the differences in exon length and total read count between samples, we further processed the data through FPKM (reads/(fragments) per kilobase of exon model per million mapped reads) and TPM (transcripts per kilobase of exon model per million mapped reads) (1 and 2).

(1) $$\mathrm{FPKM}=\frac{\mathrm{total}\;\mathrm{exon}\;\mathrm{Fragments}}{\mathrm{mapped}\;\mathrm{reads}\left(\mathrm{millions}\right)\times \mathrm{exon}\;\mathrm{length}\left(\mathrm{KB}\right)}$$

(2) $$\mathrm{TPM}=\frac{\frac{\mathrm{Ni}}{\mathrm{Li}}*10^{6}}{\mathrm{sumN}1/L1+N2/L2+\ldots +\mathrm{Nn}/\mathrm{Ln}}$$

### 4.9. GO and KEGG Enrichment Analysis

DAPs and DEGs were mapped to the database Gene Ontology to match related items. Then we used hypergeometric test (3) to find GO (gene ontology) entries that are significantly enriched in DAPs and DEGs compared with all protein backgrounds. Moreover, we analyzed the DAPs and DEGs enriched KEGG pathway by software KOBAS.

(3) $$P=1-\sum_{i-0}^{m-1}\frac{\left(\begin{array}{c} m\\ i \end{array}\right)\left(\begin{array}{cc} N & -M\\ n & -i \end{array}\right)}{\left(\begin{array}{c} N\\ n \end{array}\right)}\;$$

## 5. Conclusions

In conclusion, our results integrated the transcriptome and proteome and found the changes in genes and proteins related to the DW871 dwarf phenotype. DEGs and DAPs are mainly involved in pectin degradation, carotenoid biosynthesis, lipid metabolism, and plant hormone signal transduction. These genes are involved in lignin synthesis, pectin-lignin assembly, vascular bundle development, and regulating the component and structure of stalk tissue. DW871 is shorter than HW871 but has a longer inflorescence segment, and their stalk xylem is significantly different. These results suggest that increasing the rate of lignin monomer synthesis and changing cell wall modification not only strengthen stalk tissue structure but also limits xylem cell longitudinal extension, eventually dwarfing DW871. XETs are coregulated by auxin and BRs, and changes in XET expression result in changes in the number, morphology, and arrangement of cells in stem xylem. Our results show that auxin and BRs may be the root cause of DW871 dwarfing and that a decrease in signal strength directly influenced the expression of genes involved in lignin synthesis and cell wall modification, such as PE, LOXs, PHI1, PAL, and HCT. Dwarfing is caused directly by changes in these genes.

## Figures and Tables

**Table 1:** Plant height and inflorescence length (cm).

	0 Day		10 Days		20 Days	
Name	Height	Inflorescence Length	Height	Inflorescence Length	Height	Inflorescence Length
DW871	78.16	22.40	93.48	38.92	94.29	41.96
HW871	121.28	4.48	167.25	10.86	187.48	26.25

**Table 2:** Associated DEGs and DEPs.

Gene ID	Gene LogFC	Trends	Protein	Protein FC	Trends	Annotation
*BnaA01g06750D*	−1.97	−	A0A078GSK1	−1.03	−	XET (xyloglucan endotransglucosylase)
*BnaA01g12890D*	4.89	+	A0A078HRM3	1.63	+	Thaumatin
*BnaA01g29470D*	−1.60	−	A0A078HNZ7	−1.62	−	PME (pectinesterase)
*BnaA01g29630D*	−10.87	−	A0A078HQQ0	−1.37	−	SEP (serine-type endopeptidase)
*BnaA01g30570D*	−5.51	−	A0A078IND0	−2.63	−	CEI (cysteine proteinase inhibitor)
*BnaA02g06620D*	3.46	+	A0A078F771	1.07	+	RNA binding
*BnaA02g07120D*	−2.06	−	A0A078H1X7	−1.02	−	nsLTPs (Non-specific lipid-transfer protein)
*BnaA02g21660D*	−6.92	−	A0A078GFP1	−1.10	−	Thaumatin
*BnaA03g01600D*	−2.20	−	A0A078FEF1	−1.02	−	AEP (aspartate endopeptidase)
*BnaA04g25170D*	−9.60	−	A0A078GY31	−1.45	−	SD (Scorpion defensin)
*BnaA05g14890D*	−3.04	−	A0A078IKF2	−1.06	−	BG1 (beta-D-glucopyranosyl abscisate beta-glucosidase)
*BnaA06g34320D*	−2.61	−	A0A078FZ73,	−1.08	−	DES (Desmin)
*BnaA06g34860D*	5.06	+	A0A078FY18	1.07	+	Bet v I domain
*BnaA07g24880D*	−7.00	−	A0A078F364	−1.46	−	LOX (Lipoxygenase)
*BnaA09g07410D*	5.06	+	A0A078FZP5	1.65	+	SEP (serine-type endopeptidase)
*BnaA09g07440D*	6.58	+	A0A078G053	1.31	+	SEP (serine-type endopeptidase)
*BnaA10g27610D*	−1.86	−	A0A078HR38	−1.07	−	AAT (amino acid transport)
*BnaAnng08670D*	3.04	+	A0A078IAL2	−1.31	−	AEP (aspartic-type endopeptidase)
*BnaAnng37980D*	−10.47	−	A0A078K1B8	−1.55	−	Possible response to low-temperature stress protein
*BnaAnng41520D*	13.58	+	A0A078JY17	1.15	+	AEP (aspartic-type endopeptidase)
*BnaC02g00140D*	−15.93	−	A0A078I6N2	−2.22	−	AEP (aspartic-type endopeptidase)
*BnaC02g00890D*	−12.93	−	A0A078HE09	−2.72	−	PHI1 (phosphate-induced protein 1)
*BnaC02g09500D*	−5.99	−	A0A078FYW5	−1.05	−	FAR (Fatty acyl-CoA reductase)
*BnaC03g30640D*	−1.66	−	A0A078JWE9	−1.05	−	Plant vascular development related
*BnaCnng71290D*	−1.97	−	A0A078GSK1	1.05	+	ER transmembrane protein

**Table 3:** Genes related to monolignol synthesis and their expression patterns in *Brassica napes* L. DW871.

Encoding Enzyme	Gene Name	EC Number	Number of Genes and Trend
Phenylalanine ammonia-lyase	*PAL*	4.3.1.24	3;1 up, 2 down
Trans-cinnamate 4-monooxygenase	*CYP73A*	1.14.14.91	3;0 up, 3 down
4-coumarate--CoA ligase	*4CL*	6.1.2.12	1;0 up, 1 down
Shikimate O-hydroxycinnamoyltransferase	*HCT*	2.3.1.133	3;0 up, 3 down
Cinnamoyl-CoA reductase	*CCR*	1.2.1.44	2;0 up, 2 down
Cinnamyl-alcohol dehydrogenase	*CAD*	1.1.1.195	2;0 up, 2 down
Caffeic acid 3-O-methyltransferase	*COMT*	2.1.1.68	4;1 up, 3 down
Caffeoyl-CoA O-methyltransferase	*CCoAOMT*	2.1.1.104	4;1 up, 3 down

## Data Availability

All data are presented in this report.
